# Genetic diversity and population structure of *Glossina pallidipes *in Uganda and western Kenya

**DOI:** 10.1186/1756-3305-4-122

**Published:** 2011-06-28

**Authors:** Johnson O Ouma, Jon S Beadell, Chaz Hyseni, Loyce M Okedi, Elliot S Krafsur, Serap Aksoy, Adalgisa Caccone

**Affiliations:** 1 Trypanosomiasis Research Centre, Kenya Agricultural Research Institute, P.O. Box 362 - 00902, Kikuyu, Kenya; 2 Department of Ecology and Evolutionary Biology, Yale University, New Haven, Connecticut, USA; 3 National Livestock Resources Research Institute, Tororo, Uganda; 4 Department of Entomology, Iowa State University, Ames, Iowa, USA; 5 Yale School of Public Health, Yale University, New Haven, Connecticut, USA

## Abstract

**Background:**

*Glossina pallidipes *has been implicated in the spread of sleeping sickness from southeastern Uganda into Kenya. Recent studies indicated resurgence of *G. pallidipes *in Lambwe Valley and southeastern Uganda after what were deemed to be effective control efforts. It is unknown whether the *G. pallidipes *belt in southeastern Uganda extends into western Kenya. We investigated the genetic diversity and population structure of *G. pallidipes *in Uganda and western Kenya.

**Results:**

AMOVA indicated that differences among sampling sites explained a significant proportion of the genetic variation. Principal component analysis and Bayesian assignment of microsatellite genotypes identified three distinct clusters: western Uganda, southeastern Uganda/Lambwe Valley, and Nguruman in central-southern Kenya. Analyses of mtDNA confirmed the results of microsatellite analysis, except in western Uganda, where Kabunkanga and Murchison Falls populations exhibited haplotypes that differed despite homogeneous microsatellite signatures. To better understand possible causes of the contrast between mitochondrial and nuclear markers we tested for sex-biased dispersal. Mean pairwise relatedness was significantly higher in females than in males within populations, while mean genetic distance was lower and relatedness higher in males than females in between-population comparisons. Two populations sampled on the Kenya/Uganda border, exhibited the lowest levels of genetic diversity. Microsatellite alleles and mtDNA haplotypes in these two populations were a subset of those found in neighboring Lambwe Valley, suggesting that Lambwe was the source population for flies in southeastern Uganda. The relatively high genetic diversity of *G. pallidipes *in Lambwe Valley suggest large relict populations remained even after repeated control efforts.

**Conclusion:**

Our research demonstrated that *G. pallidipes *populations in Kenya and Uganda do not form a contiguous tsetse belt. While Lambwe Valley appears to be a source population for flies colonizing southeastern Uganda, this dispersal does not extend to western Uganda. The complicated phylogeography of *G. pallidipes *warrants further efforts to distinguish the role of historical and modern gene flow and possible sex-biased dispersal in structuring populations.

## Background

*Glossina pallidipes *is a major vector of animal African trypanosomiasis. The vector has also been implicated in the transmission of Human African Trypanosomiasis (HAT). For example, the expansion of *T. b. rhodesiense *(*Tbr) *sleeping sickness beyond its traditional focus in southeastern Uganda to western Kenya in the 1950s was attributed to *G. pallidipes *[1]. The first confirmed case of *Tbr *in Kenya was reported in 1942, having spread from southeastern Uganda along the Sio River. The spread was attributed to *Glossina pallidipes *infestation in the Busia district on the Kenya-Uganda border [2]. Further evidence for involvement of *G. pallidipes *in transmission of HAT was obtained from the isolation of the *T. b. rhodesiense *parasite from *G. pallidipes *[3]

Despite its role as a vector of trypanosomiasis, the dynamics of *G. pallidipes *populations in Uganda and the extent to which these populations are linked by dispersal to western Kenya populations were hitherto unknown. Department of Lands and Survey maps produced in the late 1960s [4] indicate that *G. pallidipes *is contiguously distributed across west-central Uganda occurring in one main belt. However, GIS prediction maps show the existence of two belts in the western and southeastern part of the country [5]. The southeastern belt extends into western Kenya and is believed to have been responsible for extending the focus of *T. rhodesiense *HAT into western Kenya [2]. The western belt occupies areas around Murchison Falls in Uganda, and regions south of Lake Albert. In the first half of the 20^th ^century, *G. pallidipes *was the most abundant species in southeastern Uganda followed by *G. brevipalpis *and *G. f. fuscipes *respectively [6]. Although all three species were thought to have dispersed into the area from other places [7], no records exist on the source populations. Fluctuation of *G. pallidipes *trap densities in Uganda in the 1970s and early 1980s [8],[9], possibly due to competition between *G. pallidipes *and *G. fuscipes*, led some authors to conclude that *G. pallidipes *could have disappeared from southeastern Uganda [10]. Recently it was claimed that *G. pallidipes *had re-invaded southeastern Uganda leading to significant increases in prevalence of trypanosome infections in cattle [11]. The re-invasion hypothesis demands a deeper understanding of the dynamics of *G. pallidipes *populations in southeastern Uganda and the adjoining western Kenya fly belt.

**Figure 1:**
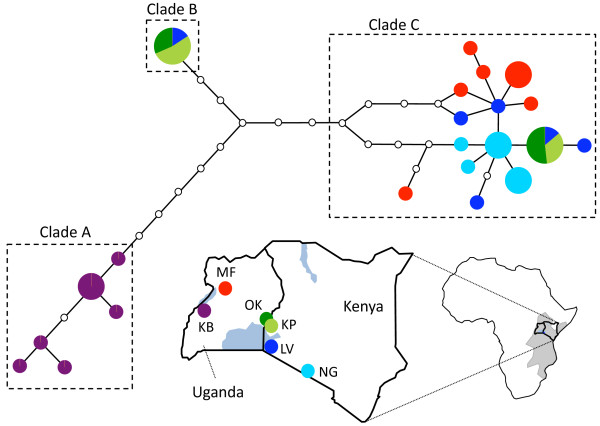
**Parsimony network of mitochondrial haplotypes**. Unique haplotypes are represented by circles, partitioned to reflect the relative frequency with which a particular haplotype was found in each of six populations of *G. pallidipes *(color coded as on map). The size of each circle is proportional to the overall frequency of that haplotype in the sample. Sampled and unsampled haplotypes (open circles) are joined by lines representing single nucleotide differences. Dotted lines delineate related groups of haplotypes (Clades A-C) referenced in the text. Populations were sampled in Kenya and Uganda in the northern portion of the range of *G. pallidipes*, which is shaded in grey.

Whereas the population structure of *G. pallidipes *in Kenya and elsewhere has been extensively studied at micro- and macrogeographic scales [12-16], no such studies have been carried out in Uganda. Furthermore, it is unclear whether the *G. pallidipes *belt in southeastern Uganda is contiguous with the western Kenya fly belt, encompassing the traditional HAT foci of Busia, Teso, and Lambwe Valley (Figure 1) as suggested earlier [17]. Here we report on the population structure of *G. pallidipes *in Uganda. In order to identify the source of tsetse in southeastern Uganda and to evaluate the extent which proposed fly belts form discrete units, we evaluated the connectivity between Ugandan populations and populations sampled at Kapesur and Lambwe Valley in western Kenya, and from Nguruman in southwestern Kenya.

## Methods

### Sampling locations and tsetse trapping

Figure 1 depicts the geographical distribution of sampled populations. *G. pallidipes *samples were collected from three locations in Uganda in 2008: Kabunkanga (KB) and Murchison Falls (MF) in western Uganda, and Okame (OK) in southeastern Uganda. Samples were also collected in Kenya from Kapesur (KP), in 2009, and Lambwe Valley (LV) and Nguruman (NG), in 2003. Sites in Lambwe and Nguruman areas were previously described [14]. Collection dates and sampling coordinates are reported in Table 1. Tsetse flies were trapped using biconical traps baited with cow urine and acetone [18]. Samples were morphologically identified as *G. pallidipes*, their sex was determined, and individual flies were stored in 85% ethanol, transferred to the laboratory and stored at -20°C until DNA extraction.

**Table 1:** Sample sizes and genetic diversity indices for mitochondrial *COI *and seven microsatellite loci in six populations of *G. pallidipes*

		Sampling	GPS	mtDNA				Microsatellites				
				
Location	Code	date	coordinates	N	# Haplotypes	H_d_	π	N	A_R _^a^	N_a_	H_o_	H_e_
**Kenya**												
Kapesur	KP	April 2003	0.7319 S; 34.31608 E	20	2	0.526	0.01439	48	3.4	21	0.488	0.471
Lambwe Valley	LV	April 2003	-0.6118 S; 34.30044E	18	6	0.856	0.01136	48	5.6	32	0.534	0.548
Nguruman	NG	April 2003	1.88925S; 36.07639E	19	4	0.591	0.00152	47	4.4	25	0.638	0.624
**Uganda**												
Kabunkanga	KB	Sept 2008	0.9800 N; 30.5500 E	16	6	0.742	0.00353	16	4.6	26	0.625	0.634
Murchison Falls	MF	Sept 2008	2.2800 N; 31.6000 E	19	6	0.749	0.00480	48	4.8	28	0.644	0.622
Okame	OK	Sept 2008	0.5221 N; 34.1149 E	21	2	0.429	0.01171	30	3.2	20	0.487	0.490

Overall				113	26			237		152		

^a ^Allelic richness (A_R_) was calculated based on a sample size of 16 individuals.

### DNA extraction, amplification, sequencing and genotyping

Genomic DNA was extracted using the DNeasy blood and tissue kit (Qiagen) as per the manufacturer's instructions. For each population, 16 to 21 of the microsatellite-genotyped individuals (see below) were randomly selected for mtDNA typing. In the case of samples from Kabunkanga (KB), we typed all of the individuals in the sample. We amplified a 473 bp fragment of cytochrome c oxidase subunit I (COI) using the primers GpCOI_F1 (5'-GAGCCTTAATTGGAGATGATC-3') and GpCOI_R1 (5'- GATGTGCTCATACAATAAATCC-3'). Fragments were amplified in a 30 μl reaction employing 1X buffer (Applied Biosystems), 0.2 mM each dNTP (New England Biolabs), 0.6 μM primers, 2 mM MgCl_2_, 0.4 mg/mL BSA (New England Biolabs) and 0.6 units AmpliTaq Gold DNA Polymerase (Applied Biosystems) using 50 cycles and an annealing temperature of 50°C. Sequencing was performed on a 3730 × l DNA Analyzer (Applied Biosystems). Sequences were deposited in GenBank (Additional file 1, Table S1).

We also typed between 16 and 48 individuals per population at 7 polymorphic microsatellite loci: GpA19a, GpB20b, GpC26b [19], GpB115 [20], GpCAG133 [21], GmC17 and GmK06 [22]. Primer sequences for the first five loci were modified with the addition of a 4 base "pigtail" sequence (GTTT) to the 5' end of the reverse primer [23]. We performed PCR using a touchdown protocol employing decreasing annealing temperatures of 61 to 51°C over 11 cycles, followed by annealing at 50°C for 35 cycles. The reactions were performed in 12.5 μl volumes using 1× PCR Buffer and 2 mM MgCl_2_, 0.2 mM each dNTP and 3 μg BSA, 5 pmol fluorescently-labeled forward primer and 5 pmol reverse primer, and 0.5 units AmpliTaq Gold.

### Descriptive statistics and marker validation

For mtDNA, we calculated haplotype diversity (*H_d_*) and nucleotide diversity (π) using the program DnaSP v5 [24]. For microsatellites, we calculated allelic richness, as well as observed (*H_o_*) and expected heterozygosities (*H_e_*), using the program GenAlex v. 6.41 [25]. Loci were tested for deviations from Hardy Weinberg equilibrium (HWE) and for linkage disequilibrium (LD) using the program Genepop v4.0 [26]. Markov chain parameters were set to 10,000 dememorizations, 1000 batches, and 10,000 iterations per batch for both tests.

### Population differentiation and structure

For both mtDNA and microsatellite data, we calculated estimates of pairwise differentiation between populations using the program Arlequin v3.1 [27] and tested for significant differentiation using 1000 permutations. We employed measures of differentiation based on haplotype or allelic frequencies (F_ST_) and measures that accounted for the evolutionary distance between haplotypes or alleles (Φ_ST_, R_ST_). Unlike F_ST_, Φ_ST _and R_ST _take into account the evolutionary distance among alleles rather than only their frequencies. We also performed an analysis of molecular variance (AMOVA) on both mtDNA and microsatellite data to evaluate the extent to which genetic variation was explained by differences among and within populations.

We evaluated evolutionary relationships among maternal (mtDNA) tsetse lineages using a parsimony network generated by the program TCS v1.21 [28]. Finer scale structuring at microsatellite loci was assessed using the Bayesian model-based clustering algorithm implemented in STRUCTURE 2.2 [29]. STRUCTURE assigns individuals to K populations based on their multilocus genotypes. We conducted five independent Markov chain Monte Carlo (MCMC) assignment runs for each K from K = 1 to K = 6 assuming an admixture model with correlated allele frequencies. We conducted the MCMC runs using 250,000 steps after throwing away the first 50,000. Evanno's criterion [30] and the method of Pritchard et al. [29] were used to identify the likeliest number of clusters. For final assignment of individuals to clusters, we used 500,000 MCMC steps. As an alternative approach for summarizing microsatellite variation across populations, we performed principle components analysis (PCA) using the "adegenet" package in R [31]. In contrast to the Bayesian assignment algorithm above, the PCA approach does not make any assumptions about HWE or LD and allows for a visual assessment of the degree to which populations differ from each other.

### Sex-specific dispersal

Microsatellite data were used to obtain pairwise genetic distance and relatedness values between individuals for all flies collected in 2008 in Uganda (KB: 4 males, 12 females; MF: 9 males, 39 females; and OK: 4 males, 26 females). Genetic distances were calculated based on [32] by using the program Alleles in Space 1.0 [33], both within and between populations, while pairwise relatedness was computed in Kingroup v2 [34] via maximum-likelihood estimation [35]. Means, standard errors, and 95% confidence intervals were determined for males and females separately. The significance of within- and between-population differences between the two sexes was tested using one-sided t-tests. Only the 2008 samples were considered in order to avoid temporal fluctuations and afford a snapshot of sex-specific dispersal patterns.

## Results

### Microsatellite validation

Following sequential Bonferroni correction, we detected no significant linkage between any pair of loci in any of the six populations. We detected a significant departure from HWE in locus *GpB20b *in three populations (Additional file 2, Table S2). Only Kapesur exhibited a significant departure after Bonferroni correction and the absolute value of F_IS _in this population was close to zero. Therefore, all further analyses included *GpB20b*.

### Genetic diversities

Among 113 *Glossina pallidipes*, twenty-two mitochondrial haplotypes were detected of which only two (# 7, 17) were shared among sampling sites, Okame, Kapesur, and Lambwe (Table 1 and Additional file 1, Table S1). Twenty mitochondrial haplotypes were 'private' (i.e., confined to a single sampling site). Nine haplotypes were singletons. Mitochondrial diversity, the probability that two randomly chosen haplotypes differ, varied from 0.43 in Okame to 0.86 in Lambwe. The overall mean was 0.65 ± 0.15. Nucleotide diversities π (the average number of nucleotide differences per site) varied nearly ten-fold, from only c. 0.0015 in Nguruman to 0.014 at Kapesur and an overall mean of 0.008 ± 0.005.

Among 237 flies (474 genomes), seven microsatellite loci afforded 51 alleles (Table 1). The number of alleles per locus ranged from 2 at *GmC17 *to 18 at *GpB20b*. Average allelic richness (A_R_, allelic diversity corrected for variations in sample size) ranged from 3.2 to 5.6, and was least in Okame and Kapesur; these locations also exhibited the least heterozygosities although *He *values did not differ significantly from the four other estimates. Microsatellite diversity measures for Lambwe were not significantly greater than in Okame and Kapesur (*H*_(df = 2) _= 0.27, P = 0.87 ). The foregoing locations share the same fly belt but Lambwe *G. pallidipes *has been subjected to repeated, and unsuccessful eradication attempts in the past 30 years [36].

Estimates of the expected (*He*) and observed (*Ho*) microsatellite diversities were closely similar, thereby roughly indicating random matings within populations. Formal tests of hypothesis are provided by *F_IS _*= 0 and indicated a significant difference at only one locus in only one sample (*GpB20b *at KP, Additional file 2, Table S2). Population *F_IS_*, averaged over loci, indicated no departures from random mating within populations (Table 3).

### Genetic differentiation and population structure

AMOVA results (Table 2) confirmed that differences among populations contributed a significant proportion of the variance observed in the distribution of mtDNA haplotypes. The proportion of the variance explained by population differences was greater when accounting for the evolutionary relationships among haplotypes (Φ_ST_) than when just considering haplotype frequencies alone (*F_ST_*). Pairwise *F_ST_*s for mtDNA ranged from 0.046 (OK vs. KP and OK vs. LV) to 0.922 (KB vs. NG) while values for Φ_ST _ranged from 0.046 (OK vs. KP) to 0.493 (OK vs. NG) (Table 3). All values were significantly different from zero except for comparisons involving the neighboring populations Okame (OK), Kapesur (KP), and Lambwe Valley (LV), indicative of substantial gene flow among them.

**Table 2:** Results of an AMOVA testing for genetic structure in *G. pallidipes *sampled in Kenya and Uganda

	d.f	Sum of squares	Variance components	% Variation	p
**mtDNA (*F*_ST_)**					
Among sites	5	15.33	0.146	31.4	0.000
Within sites	107	34.18	0.319	68.6	
**mtDNA (*Φ*_ST_)**					
Among sites	5	204.44	2.078	53.5	0.000
Within sites	107	193.24	1.806	46.5	
**microsatellites (*F*_ST_)**					
Among sites	5	233.66	0.582	26.5	0.000
Within sites	468	757.62	1.619	73.5	
**microsatellites (*R*_ST_)**					
Among sites	5	19793.83	48.573	19.8	0.000
Within sites	468	91978.08	196.534	80.2	

Results for microsatellites represent the weighted average over 7 loci. *F*_ST _reflects differences in haplotype (mtDNA) or allele (microsatellites) frequencies while *Φ*_ST _accounts for the evolutionary relatedness of those haplotypes or alleles.

**Table 3:** Estimates of *F*_ST _at the mitochondrial locus *COI *for populations of *G. pallidipes*

	KB	MF	OK	KP	LV	NG
KB	-	**0.867**	**0.732**	**0.671**	**0.752**	**0.922**
MF	**0.255**	-	**0.349**	**0.429**	**0.189**	**0.472**
OK	**0.426**	**0.415**	-	0.046	0.046	**0.306**
KP	**0.372**	**0.364**	0.046	-	0.151	**0.446**
LV	**0.200**	**0.198**	0.197	0.145	-	**0.188**
NG	**0.337**	**0.330**	**0.493**	**0.442**	**0.278**	-

*F*_ST _was calculated using pairwise distances (above diagonal) and haplotype frequencies (below diagonal). Comparisons that were significant after Bonferroni correction (p < 0.003) are indicated in bold type.

As evident in a haplotype network (Figure 1 and additional file 1, Table S1), only two of the 23 haplotypes were shared between any populations. These two haplotypes were the only sequences recovered from flies in Okame (OK) and Kapesur (KP) and represented a subset of the haplotypes found in flies from Lambwe Valley (LV). One of these haplotypes is found within a clade of widespread maternal lineages (Clade C), while the other is the only representative of Clade B. Clades A and B are quite distant from each other and from Clade A (2.4% and 3.3% of constituent nucleotides, respectively), although both clades include haplotypes from sampling sites that are geographically close to sites where only clade C haplotypes are recovered. Clades A and B topologies are also very different from the one for clade C. Clades A and B include either only one (Clade B) or a few (Clade A) recently diverged haplotypes, as suggested by the small number of mutational steps that separate them. Clade C not only comprises more than twice as many haplotypes than clade A, it includes haplotypes found in the same population which are separated by more mutational steps from haplotypes found in the same populations than haplotypes only found in other populations.

**Figure 2:**
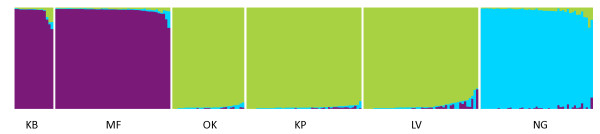
**Plot of Bayesian assignment probabilities based on microsatellite genotypes**. Vertical bars are shaded to indicate the probability of assignment of an individual genotype to one of three clusters (black, dark grey, light grey) inferred by STRUCTURE. Within each population (separated by a white line and identified at the bottom), individuals have been sorted by decreasing membership in the cluster with highest assignment probability for that population.

**Figure 3:**
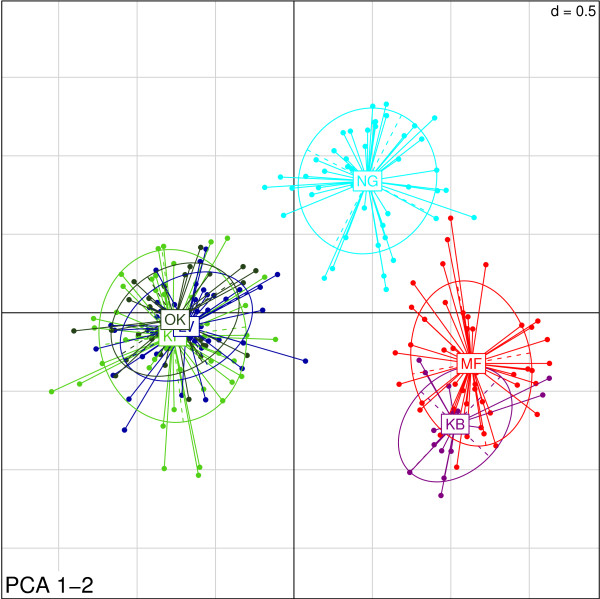
**Genetic structure inferred from principal component analysis on microsatellite variation in *G. pallidipes***. Points represent individual genotypes sampled from a particular population and are connected by lines to the 95% confidence ellipse centroid of the respective population.

Microsatellite data revealed three clusters of genetically distinct populations which were consistent with the patterns of genetic differentiation indicated by mitochondrial DNA with the notable exception of flies from Murchison Falls. In contrast to the large pairwise distance observed between Murchison Falls (MF) and Kabunkanga (KB) in mtDNA (*F_ST _*= 0.255, Φ_ST _= 0.867; Table 3), measures of differentiation based on microsatellites (*F_ST _*= 0.056, *R_ST _*= 0.009; Table 4) were much smaller and similar to those observed among the neighboring populations Okame, Kapesur and Lambwe Valley, which ranged from 0.022 to 0.085 for *F_ST _*and 0.021 to 0.033 for *R_ST _*(Table 4). Bayesian clustering of individual microsatellite-based genotypes indicated the presence of three distinct clusters corresponding to geographically proximate sampling locations (Figure 2). These clusters consisted of flies from: Kabunkanga (KB) and Murchison Falls (MF); Okame (OK), Kapesur (KP) and Lambwe Valley (LV); and Nguruman (NG). We observed no evidence of admixture of flies between these three genetically and geographically distinct clusters. Principle components analysis (Figure 3) indicated that tsetse flies from Nguruman were as genetically distinct from neighboring flies in Okame/Kapesur/Lambwe Valley as they were from tsetse sampled further away in western Uganda (Kabunkanga/Murchison Falls). The first two axes of the PCA explained approximately 40% of the total variance.

**Table 4:** Estimates of *F*_ST _(below diagonal) and *R*_ST _(above diagonal) and *F*_IS _on diagonal in *G. pallidipes *measured across seven microsatellite loci

	KB	MF	OK	KP	LV	NG
KB	**0.024**	0.009	0.311	0.312	0.388	0.414
MF	0.056	**-0.046**	0.198	0.194	0.259	0.253
OK	0.387	0.354	**-0.020**	0.023	0.033	0.214
KP	0.369	0.357	0.022	**0.001**	0.021	0.173
LV	0.367	0.338	0.076	0.085	**0.013**	0.152
NG	0.304	0.215	0.283	0.294	0.281	**-0.030**

Comparisons that were significant after Bonferroni correction (p < 0.003) are indicated in bold type.

### Sex-specific dispersal

**Figure 4:**
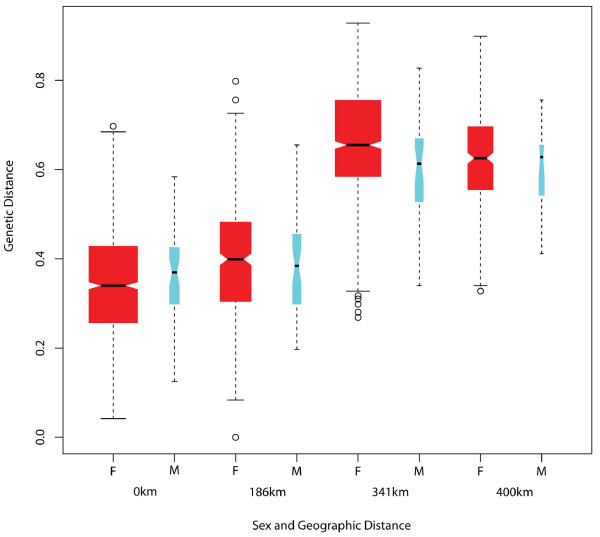
**Box plots of Genetic Dissimilarity Grouped by Sex and Geographic Distance Class.** Boxplots with 95% confidence intervals (including outliers, shown as circles) of pairwise genetic distances between individuals, grouped by distance class for within (0 km, Kabunkanga, Murchison Falls, and Okame) and between populations (186 km: Kabunkanga-Murchison Falls; 341 km: Murchison Falls-Okame; 400 km: Kabunkanga-Okame) and sex (F - female, M - male).

We used microsatellite data collected from three localities (KB, MF and OK), where flies were sampled in the same season (March-April 2008), to determine whether mobility differs significantly between sexes. Table 5 and Figure 4 show the results. Mean pairwise relatedness was significantly higher in females than in males within populations. Between-population comparisons in the KB-MF-OK triangle (approximately 35,000 km^2^) showed greater genetic similarity between males than females. The lack of significance in one comparison, between KB and OK (341 km apart), is likely due to small male sample sizes at both sites.

**Table 5:** Relatedness and genetic distance values were obtained from microsatellite data and computed both within and between samples (KB - Kabunkanga, MF - Murchison Falls, OK - Okame)

Geographic Distance	Genetic Distance Between Individuals				Relatedness Between Individuals			
	**dF**	**dM**	**Hypothesis**	***P*-value (t-test)**	**rF**	**rM**	**Hypothesis**	***P*-value (t-test)**

*0 km (within: KB, MF, OK)*	0.344	0.359	dF < dM	0.176	0.240	0.144	rF > rM	***0.008***
*186 km (between: KB, MF)*	0.399	0.383	dF > dM	0.208	0.123	0.115	rF < rM	0.584
*341 km (between: MF, OK)*	0.658	0.599	dF > dM	***0.002***	-0.141	-0.078	rF < rM	***0.008***
*400 km (between: KB, OK)*	0.629	0.602	dF > dM	0.145	-0.144	-0.121	rF < rM	0.208
*186-400 km (between: KB, MF, OK)*	0.585	0.511	dF > dM	***0.000***	-0.073	-0.007	rF < rM	***0.002***

dF - mean pairwise genetic distance between female flies, dM - mean pairwise genetic distance between male flies, rF - mean pairwise relatedness between females, rM - mean pairwise relatedness between males.

One-sided t-tests were used to ascertain the significance of male-biased dispersal, i.e. higher relatedness and lower genetic distance of male versus female individuals between populations. Significant P-values are denoted in bold, italic font

## Discussion

All studies to date of breeding structure in the Morsitans group tsetse flies have indicated highly structured populations among which there has been little detectable gene flow [16]. Our results in *G. pallidipes *are in agreement with the earlier findings. Populations in western Uganda were significantly differentiated from flies in the northeastern corner of Lake Victoria, and these populations were further differentiated from the population in Nguruman in south-central Kenya. Furthermore, tsetse populations were not homogeneous within the three regions.

Indices of differentiation inferred from mtDNA and microsatellites indicated that populations at Okame, Kapesur and Lambwe Valley form a genetically homogeneous group relative to the populations lying approximately 400 km to the east or west. Within this group, however, genetic diversity was less in Okame and Kapesur than in Lambwe Valley. In fact, mtDNA haplotypes recovered from Okame and Kapesur formed a subset of those found in the Lambwe Valley. Similarly, with the exception of one allele at one locus, microsatellite alleles in Okame and Kapesur were also a subset of those found in the Lambwe Valley (data not shown). Past control operations under the Farming in Tsetse Controlled Areas (FITCA) project http://www.au-ibar.org/index.php/en/projects/completed-projects/fitca/achievements, are likely to be responsible for the genetic structuring. Historically, the three populations may have been part of a large, panmictic, and genetically diverse population, and control activities may have severely reduced population sizes in Okame and Kapesur leading to the observed reduction in genetic diversity. Once the FITCA project ended in the early 2000s, gene flow from Lambwe Valley could have led to increased genetic diversity and allelic homogenization. Alternatively, the Okame and Kapesur populations are not relicts of a larger population but originated from two recent colonizations from the Lambwe Valley. *A priori*, both scenarios are equally likely. However, since earlier genetic studies indicated that the Lambwe Valley tsetse population is large and has been in residence for a long time [14], it is most likely that the low genetic diversity observed in Okame and Kapesur flies is due to recent colonization rather than a past bottleneck.

As in the Lake Victoria region, populations in western Uganda differed significantly over the approximately 190 km separating Kabunkanga and Murchison Falls. Populations of *G. pallidipes *at Kabunkanga and Murchison Falls exhibited similar microsatellite frequencies, but extremely divergent mtDNA haplotypes.

Because of differences in evolutionary rates and inheritance patterns between bi-parentally inherited microsatellite loci and maternally-inherited mtDNA, direct comparisons between the results of these two types of molecular marker might be misleading. To investigate the possibility of sex-biased dispersal, in addition to comparing microsatellite and mtDNA results, we carried out an individual-based sex-specific analysis of the level of genetic differentiation and relatedness using only microsatellite data. If dispersal is sex-biased, we expect to encounter higher genetic differentiation within populations and more genetically similar individuals across populations in the better-dispersing sex, while the more philopatric sex will exhibit higher relatedness values between individuals within populations and increased genetic dissimilarity and lesser relatedness between populations relative to the more mobile sex [37].

Our data can suggest that males disperse over longer distances than females (Table 5 and Figure 4). Despite the fact that females are believed to be highly mobile [38] due to their relatively larger body size, males are active for longer periods of time [39] and devote blood-meals exclusively to the production of fat, which is used as an energy reserve for flight [40]. Additionally, the asymmetry in male versus female dispersal could be attributed to flight constraints imposed on females by carrying a larva, which can double the weight of a female at the peak of pregnancy [41]. The male-biased dispersal recovered from microsatellite data needs further scrutiny as the small male sample sizes in this study did not allow for rigorous testing of hypothesis, as the study was not designed for this purpose.

The low level of microsatellite differentiation between tsetse at Kabunkanga and Murchison Falls is also hard to reconcile with the absolute divergence in *COI *sequences observed between tsetse flies from these two sites. The net average nucleotide divergence was 2.7%, consistent with a divergence time of 1.8 million years, assuming a molecular clock ticking at 1.5% divergence per million years [33]. Therefore, unequal dispersal rates would have to have been maintained for an extremely long period in order to generate the conflicting signals in microsatellites and mtDNA.

A mitochondrial sweep, due perhaps to *Wolbachia *infection favoring the amplification of a particular mitochondrial lineage in one population, could have shortened the time frame over which this apparent divergence accumulated. Even in this case, though, sufficient time has passed to allow the accumulation of mtDNA diversity in both populations without any concomitant exchange of haplotypes. Owing to the possibility of past bottlenecks and rare long-distance colonizations, as well as sex-biased dispersal, the phylogeographic history of *G. pallidipes *appears to be complex.

Aside from the seemingly contradictory signals from microsatellites and mtDNA in western Uganda, we also observed neighboring populations in the Lake Victoria region that shared two mtDNA lineages differing by about 2% without observing any of the intervening haplotypes. This would suggest that, *G. pallidipes *colonized the Lake Victoria region independently at least twice or a very large and diverse population of *G. pallidipes *underwent a severe bottleneck or series of lesser bottlenecks, leaving only remnants of the past diversity. A deeper understanding of the phylogeography of *G. pallidipes *will require greater context and range-wide relationships should be explored more thoroughly in the future.

The current study greatly enhances our understanding of *G. pallidipes *population dynamics especially in Uganda, which has been a missing link in previous samplings. To the best of our knowledge, this is the first report on the population structure of this species in Uganda based on natural samples. In an earlier paper that described the population structure of *G. pallidipes *at a macrogeographic scale covering almost its entire range, only a single sample from a laboratory colony of *G. pallidipes *originating from Uganda nearly three decades ago was analyzed [15]. In another study Ouma et al. [14] discussed the relict *G. pallidipes *populations in Lambwe and Nguruman, and demonstrated temporal and seasonal stability of *G. pallidipes *populations in these areas. Such temporal stability has also been reported in *G. fuscipes fuscipes *[42]. These previous studies were reviewed [16] and suggested significant differentiation among natural populations of *G. pallidipes *in eastern and southern Africa. However, in the absence of samples from Uganda, it was always difficult to put the data into perspective and understand the re-infestation of western Kenya including Lambwe Valley and Busia-Teso regions by *G. pallidipes*.

The findings of this study have reaffirmed the importance of gathering genetic data prior to implementing area-wide tsetse vector control operations as recommended for creation of *G.p. gambiensis *free zones in the Niayes region of Senegal [43]. Genetic data should be generated as part of baseline data collection to provide the much needed scientific evidence upon which control measures can be effectively implemented.

## Conclusion

This study underscores the importance of tailoring both monitoring and control measures to the population-specific circumstances and history, and the importance of understanding the evolutionary dynamics likely to have shaped the breeding structure of each population. This is exemplified by our findings at different levels:

1- On a broad spatial scale our results point to the presence of at least three genetically discrete fly belts among which there has been little detectable gene flow in the region extending from western Uganda to Nguruman in southwestern Kenya. Such strong geographic structuring of *G. pallidipes *should limit the geographic scale on which area wide vector control needs to be implemented.

2- On a local scale our data point to specific populations where control and detection methods need improvement. In keeping with earlier studies [14], [16], we have identified the Lambwe Valley as a region where such revision is needed for two reasons. First, despite years of intensive control efforts and very low fly densities detected by current trapping methods, the population is still highly variable genetically and thus probably quite large. Second, this population has served as a source for seeding neighboring regions.

3- Our data suggest the existence of both historical (mtDNA-microsatellite comparion) and current (microsatellite-inferred) male-biased dispersal. This contradicts the general idea that females are better dispersers than males and because of its relevance for control and eventual sterile insect release activities should be further explored to understand its genetic, ecological and physiological underpinnings. Further research is needed to clarify sex-biased dispersal in this species and to demonstrate it in other morsitans group flies.

4- Finally, control efforts on small populations may vary in efficacy and can be optimized if coupled with inferences from genetic data, as exemplified by Okame/Kapesur. If flies from these sites originated as rare immigrations from neighboring sites, as we suggest, control efforts can be long-lasting, even if control measures and monitoring activities are lessened over time, as the probability of re-infestation is low, as shown by extensive studies on breeding structure in morsitans group flies. On the other hand, if increases in tsetse densities in a given area are due to expansion of relict populations rather than re-infestation, then efforts can be ineffective, if local control is lessened before the population is completely extirpated. This task is rather difficult not only to achieve but also to evaluate by using traditional sampling methods.

## Competing interests

The authors declare that they have no competing interests.

## Authors' contributions

JOO conceived the paper, performed the wet lab analyses, and wrote the first version of the manuscript. ESK, AC, SA, JSB and CH revised the manuscript. CH and JSB performed wet lab and statistical analyses and helped in the interpretation of results. LMO provided background and ecological information that helped with study design. LMO and JSB collected samples in Uganda. ESK and JOO did the 2003 samplings. SA and AC jointly supervised the work at Yale University and helped conceive the paper. All authors read and approved the final manuscript.

## Supplementary Material

Additional file 1 **Table S1**. *Cytochrome oxidase I *in *Glossina pallidipes*: frequencies of haplotypes observed across populations and associated GenBank accession numbers. Click here for file

Additional file 2 **Table S2**. Estimates of *F*_IS _at 7 microsatellite loci for populations of *G. pallidipes*. Significance was assessed at p < 0.05 (*) and, after Bonferroni correction, at p < 0.008 (**). Click here for file
