# Supplementary material for: Genetic diversity and population structure of Glossina pallidipes in Uganda and western Kenya
Source: Parasit Vectors. 2011 Jun 28;4:122. doi: 10.1186/1756-3305-4-122 (PMC3146932; doi:10.1186/1756-3305-4-122)
Supplement: Additional file 1 — Table S1. Cytochrome oxidase I in Glossina pallidipes: frequencies of haplotypes observed across populations and associated GenBank accession numbers. [file 1756-3305-4-122-S1.DOC]

**Table S1.** *Cytochrome oxidase I* in *Glossina pallidipes*: frequencies observed across populations and associated GenBank accession numbers.

| Haplotype ID | Population | Frequency | GenBank no. |
| --- | --- | --- | --- |
| 1 | Kabunkanga | 1 | TBD |
| 2 | Kabunkanga | 5 | TBD |
| 3 | Kabunkanga | 1 | TBD |
| 4 | Kabunkanga | 7 | TBD |
| 5 | Kabunkanga | 1 | TBD |
| 6 | Kabunkanga | 1 | TBD |
| 7 | Okame | 6 | TBD |
| 7 | Kapesur | 10 | TBD |
| 7 | Lambwe Valley | 3 | TBD |
| 8 | Murchison Falls | 1 | TBD |
| 9 | Murchison Falls | 3 | TBD |
| 10 | Murchison Falls | 2 | TBD |
| 11 | Murchison Falls | 9 | TBD |
| 12 | Lambwe valley | 2 | TBD |
| 13 | Lambwe valley | 4 | TBD |
| 14 | Murchison Falls | 3 | TBD |
| 15 | Nguruman | 1 | TBD |
| 16 | Nguruman | 11 | TBD |
| 17 | Okame | 15 | TBD |
| 17 | Kapesur | 10 | TBD |
| 17 | Lambwe Valley | 3 | TBD |
| 18 | Lambwe Valley | 1 | TBD |
| 19 | Murchison Falls | 1 | TBD |
| 20 | Nguruman | 1 | TBD |
| 21 | Lambwe Valley | 4 | TBD |
| 22 | Nguruman | 6 | TBD |
