# Supplementary material for: Genetic diversity and population structure of Glossina pallidipes in Uganda and western Kenya
Source: Parasit Vectors. 2011 Jun 28;4:122. doi: 10.1186/1756-3305-4-122 (PMC3146932; doi:10.1186/1756-3305-4-122)
Supplement: Additional file 2 — Table S2. Estimates of FIS at 7 microsatellite loci for populations of G. pallidipes. Significance was assessed at p < 0.05 (*) and, after Bonferroni correction, at p < 0.008 (**). [file 1756-3305-4-122-S2.DOC]

**Table S2. Estimates of *F*_IS_ at 7 microsatellite loci for populations of *G. pallidipes***. Significance was assessed at p < 0.05 (*) and, after Bonferroni correction, at p < 0.008 (**).

|  | Microsatellite Locus | | | | | | |
| --- | --- | --- | --- | --- | --- | --- | --- |
| Population | GpA19a | GpB20b | GpB115 | GmC17 | GpC26b | GpCAG133 | GmK06 |
| **KENYA** |  |  |  |  |  |  |  |
| KP | 0.2030 | -0.0050** | -0.1619 | 0.0390 | -0.1006 | NA | 0.0315 |
| LV | 0.0053 | 0.1405* | -0.0427 | -0.0227 | 0.0319 | NA | -0.0237 |
| NG | -0.0851 | 0.0331 | -0.0705 | -0.1646 | 0.0173 | 0.0590 | NA |
| **UGANDA** |  |  |  |  |  |  |  |
| KB | 0.1638 | 0.0674 | 0.0149 | NA | 0.3204 | -0.4008 | NA |
| MF | -0.0860 | 0.2193* | -0.1533 | 0.0000 | -0.0631 | -0.1686 | 0.1975 |
| OK | -0.0223 | 0.1760 | 0.0368 | -0.0545 | -0.1125 | NA | -0.1600 |
